# Poly[[aqua­(μ_2_-oxalato)(μ_2_-2-oxido­pyridinium-3-carboxylato)dysprosium(III)] monohydrate]

**DOI:** 10.1107/S1600536809000580

**Published:** 2009-01-10

**Authors:** Chun-De Huang, Jie-Xuan Huang, Yi-Yi Wu, Ying-Yang Lian, Rong-Hua Zeng

**Affiliations:** a School of Chemistry and the Environment, South China Normal University, Guangzhou 510006, People’s Republic of China; b South China Normal University, Key Laboratory of Technology on Electrochemical Energy Storage and Power Generation in Guangdong Universities, Guangzhou 510006, People’s Republic of China

## Abstract

In the title complex, {[Dy(C_6_H_4_NO_3_)(C_2_O_4_)(H_2_O)]·H_2_O}_*n*_, the Dy^III^ ion is coordinated by seven O atoms from two 2-oxidopyridinium-3-carboxylate ligands, two oxalate ligands and one water mol­ecule, displaying a distorted bicapped trigonal-prismatic geometry. The carboxyl­ate groups of the 2-oxidopyridinium-3-carboxylate and oxalate ligands link dysprosium metal centres, forming layers parallel to (100). These layers are further connected by inter­molecular O—H⋯O hydrogen-bonding inter­actions involving the coordin­ated water mol­ecules, forming a three-dimensional supra­molecular network. The uncoordinated water mol­ecule is involved in N—H⋯O and O—H⋯O hydrogen-bonding inter­actions within the layer.

## Related literature

For background to the mol­ecular self-assembly of supra­molecular architectures, see: Moulton & Zaworotko (2001 ▶); Zeng *et al.* (2007 ▶).
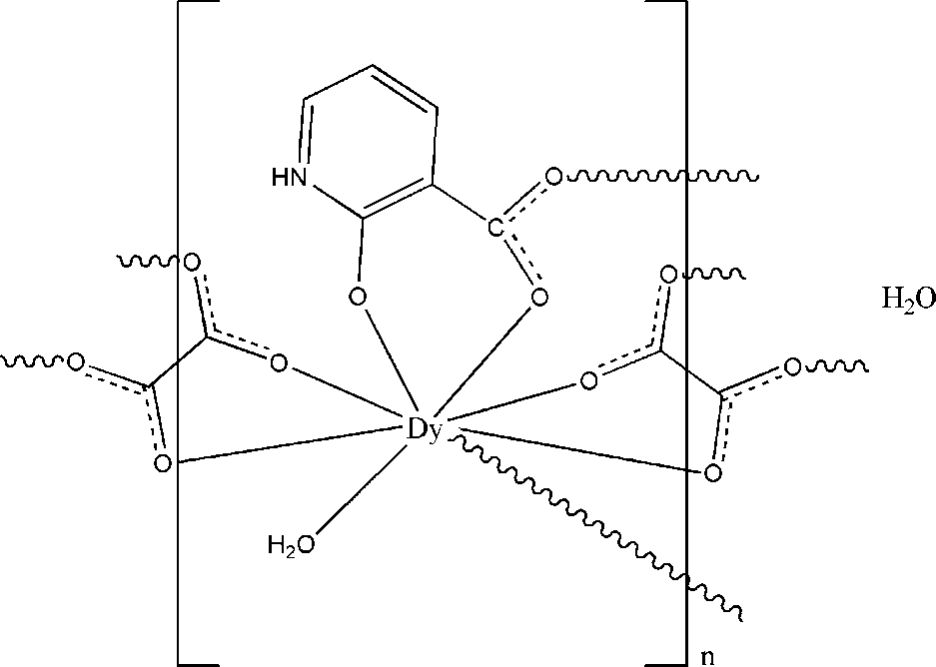


## Experimental

### 

#### Crystal data

[Dy(C_6_H_4_NO_3_)(C_2_O_4_)(H_2_O)]·H_2_O
*M* *_r_* = 424.65
Triclinic, 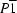

*a* = 6.5359 (15) Å
*b* = 9.561 (2) Å
*c* = 9.734 (2) Å
α = 71.906 (2)°
β = 78.800 (3)°
γ = 80.305 (2)°
*V* = 563.4 (2) Å^3^
*Z* = 2
Mo *K*α radiation
μ = 6.68 mm^−1^
*T* = 296 (2) K
0.17 × 0.16 × 0.14 mm

#### Data collection

Bruker APEXII area-detector diffractometer
Absorption correction: multi-scan (*APEX2*; Bruker, 2004 ▶) *T*_min_ = 0.397, *T*_max_ = 0.455 (expected range = 0.342–0.393)
2926 measured reflections
2003 independent reflections
1888 reflections with *I* > 2σ(*I*)
*R*_int_ = 0.021

#### Refinement

*R*[*F* ^2^ > 2σ(*F* ^2^)] = 0.030
*wR*(*F* ^2^) = 0.077
*S* = 1.09
2003 reflections
172 parameters
H-atom parameters constrained
Δρ_max_ = 2.29 e Å^−3^
Δρ_min_ = −1.55 e Å^−3^

### 

Data collection: *APEX2* (Bruker, 2004 ▶); cell refinement: *SAINT* (Bruker, 2004 ▶); data reduction: *SAINT*; program(s) used to solve structure: *SHELXS97* (Sheldrick, 2008 ▶); program(s) used to refine structure: *SHELXL97* (Sheldrick, 2008 ▶); molecular graphics: *ORTEPIII* (Burnett & Johnson, 1996 ▶) and *PLATON* (Spek, 2003 ▶); software used to prepare material for publication: *SHELXL97*.

## Supplementary Material

Crystal structure: contains datablocks I, global. DOI: 10.1107/S1600536809000580/dn2421sup1.cif

Structure factors: contains datablocks I. DOI: 10.1107/S1600536809000580/dn2421Isup2.hkl

Additional supplementary materials:  crystallographic information; 3D view; checkCIF report

## Figures and Tables

**Table 1:** Hydrogen-bond geometry (Å, °)

*D*—H⋯*A*	*D*—H	H⋯*A*	*D*⋯*A*	*D*—H⋯*A*
N1—H1⋯O2*W*	0.86	1.99	2.785 (9)	154
O1*W*—H1*W*⋯O2^i^	0.85	1.94	2.732 (7)	155
O1*W*—H2*W*⋯O4^ii^	0.85	2.07	2.751 (7)	137
O2*W*—H4*W*⋯O1^iii^	0.85	2.26	3.080 (8)	163
O2*W*—H4*W*⋯O6^iii^	0.85	2.36	2.878 (8)	120

Symmetry codes: (i) 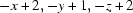; (ii) 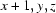; (iii) 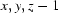.

**Fig. 1.**
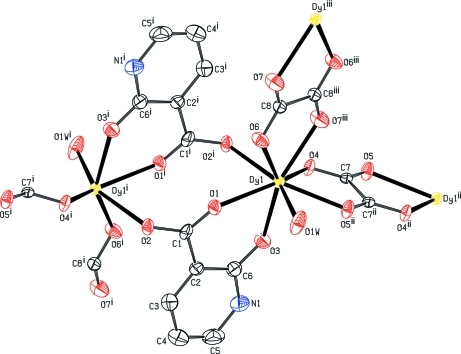
The molecular structure showing the atomic-numbering scheme. Displacement ellipsoids drawn at the 30% probability level. Symmetry codes: (i)1 - x, 1 - y, 2 - z; (ii)1 - x, 2 - y, 1 - z; (iii)1 - x, 2 - y, 2 - z.

**Fig. 2.**
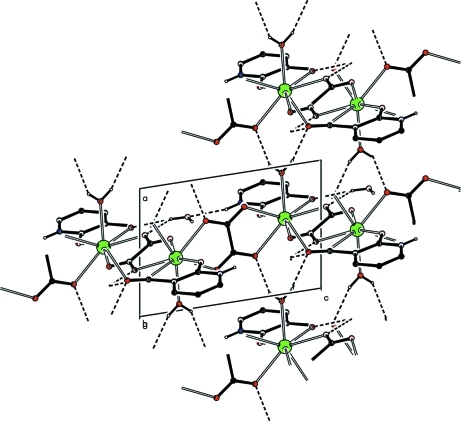
A view of the three-dimensional supramolecular network. Hydrogen bonds are shown as dashed lines.

## supplementary crystallographic information

### Comment

Molecular self-assembly of supramolecular architectures has received much attention during recent decades (Zeng *et al.*, 2007; Moulton & Zaworotko, 2001). The structures and properties of such systems depend on the coordination and geometric preferences of both the central metal ions and the bridging building blocks, as well as the influence of weaker non-covalent interactions, such as hydrogen bonds and π-π stacking interactions. Recently, we obtained the title coordination polymer, which was synthesized under hydrothermal conditions.

In the structure of the title compound, each Dy^III^ centre is in a bicapped trigonal prismatic geometry, defined by seven oxygen atoms from two 2-oxidopyridinium-3-carboxylate ligands, one oxalate ligand, and one water molecule Fig. 1. The Dy^III^ ions are linked by 2-oxidopyridinium-3-carboxylate ligands and oxalate ligands to form a layer in the *bc* plane, and the adjacent Dy···Dy separations are 5.858 (4), 6.186 (5) and 6.239 Å, respectively. The layers are further connected by ιntermolecular O—H···O hydrogen bonding interactions inolving the coordinated water molecules to form a three-dimensional supramolecular network (Table 1, Fig. 2). Within each layer, free water molecules further link the complexes  through N-H···O and O-H···O bonding interactions (Table 1).

### Experimental

A mixture of Dy_2_O_3_ (0.375 g; 1 mmol), 2-oxynicotinic acid (0.127 g; 1 mmol), oxalic acid (0.09 g; 1 mmol), water (10 ml) in the presence of HNO_3_ (0.024 g; 0.385 mmol) was stirred vigorously for 20 min and then sealed in a Teflon-lined stainless-steel autoclave (20 ml, capacity). The autoclave was heated and maintained at 446 K for 2 days, and then cooled to room temperature at 5 K h^-1^ and obtained the colorless block crystals.

### Refinement

Water H atoms were tentatively located in difference Fourier maps and were refined with distance restraints of O–H = 0.85 Å and H···H = 1.39 Å, and with *U*_iso_(H) = 1.5 *U*_eq_(O), and then were treated as riding mode. H atoms attached to C and N atoms were placed at calculated positions and were treated as riding on their parent atoms with C—H = 0.93 Å, and N-H= 0.86Å with *U*_iso_(H) = 1.2 *U*_eq_(C,N).

### Crystal data

**Table:**

[Dy(C_6_H_4_NO_3_)(C_2_O_4_)(H_2_O)]·H_2_O	*Z* = 2
*M**_r_* = 424.65	*F*(000) = 402
Triclinic, *P*1	*D*_x_ = 2.503 Mg m^−^^3^
Hall symbol: -P 1	Mo *K*α radiation, λ = 0.71073 Å
*a* = 6.5359 (15) Å	Cell parameters from 6377 reflections
*b* = 9.561 (2) Å	θ = 1.7–28.0°
*c* = 9.734 (2) Å	µ = 6.68 mm^−^^1^
α = 71.906 (2)°	*T* = 296 K
β = 78.800 (3)°	Block, colourless
γ = 80.305 (2)°	0.17 × 0.16 × 0.14 mm
*V* = 563.4 (2) Å^3^	

### Data collection

**Table:**

Bruker APEXII area-detector diffractometer	2003 independent reflections
Radiation source: fine-focus sealed tube	1888 reflections with *I* > 2σ(*I*)
graphite	*R*_int_ = 0.021
φ and ω scans	θ_max_ = 25.2°, θ_min_ = 2.2°
Absorption correction: multi-scan (*APEX2*; Bruker, 2004)	*h* = −7→7
*T*_min_ = 0.397, *T*_max_ = 0.455	*k* = −11→9
2926 measured reflections	*l* = −11→7

### Refinement

**Table:**

Refinement on *F*^2^	Primary atom site location: structure-invariant direct methods
Least-squares matrix: full	Secondary atom site location: difference Fourier map
*R*[*F*^2^ > 2σ(*F*^2^)] = 0.030	Hydrogen site location: inferred from neighbouring sites
*wR*(*F*^2^) = 0.077	H-atom parameters constrained
*S* = 1.09	*w* = 1/[σ^2^(*F*_o_^2^) + (0.0379*P*)^2^ + 2.6561*P*] where *P* = (*F*_o_^2^ + 2*F*_c_^2^)/3
2003 reflections	(Δ/σ)_max_ = 0.001
172 parameters	Δρ_max_ = 2.29 e Å^−^^3^
0 restraints	Δρ_min_ = −1.55 e Å^−^^3^

### Special details

**Table:**

Geometry. All esds (except the esd in the dihedral angle between two l.s. planes) are estimated using the full covariance matrix. The cell esds are taken into account individually in the estimation of esds in distances, angles and torsion angles; correlations between esds in cell parameters are only used when they are defined by crystal symmetry. An approximate (isotropic) treatment of cell esds is used for estimating esds involving l.s. planes.
Refinement. Refinement of *F*^2^ against ALL reflections. The weighted *R*-factor *wR* and goodness of fit *S* are based on *F*^2^, conventional *R*-factors *R* are based on *F*, with *F* set to zero for negative *F*^2^. The threshold expression of *F*^2^ > σ(*F*^2^) is used only for calculating *R*-factors(gt) *etc*. and is not relevant to the choice of reflections for refinement. *R*-factors based on *F*^2^ are statistically about twice as large as those based on *F*, and *R*- factors based on ALL data will be even larger.

### Fractional atomic coordinates and isotropic or equivalent isotropic displacement parameters (Å^2^)

**Table:**

	*x*	*y*	*z*	*U*_iso_*/*U*_eq_	
Dy1	0.59123 (5)	0.79001 (3)	0.80042 (3)	0.01511 (12)	
O4	0.3353 (7)	0.8866 (5)	0.6354 (5)	0.0200 (10)	
O1	0.7142 (8)	0.5573 (5)	0.9516 (5)	0.0228 (10)	
O7	0.5714 (9)	0.9762 (6)	1.1684 (5)	0.0332 (13)	
C6	0.7318 (12)	0.4932 (8)	0.6786 (8)	0.0255 (15)	
O3	0.6553 (9)	0.6282 (5)	0.6621 (5)	0.0276 (11)	
C8	0.5595 (11)	0.9416 (7)	1.0579 (7)	0.0205 (14)	
O6	0.6333 (8)	0.8228 (5)	1.0278 (5)	0.0278 (11)	
C7	0.3857 (10)	0.9806 (7)	0.5168 (7)	0.0162 (13)	
O5	0.2701 (8)	1.0470 (5)	0.4234 (5)	0.0243 (11)	
C1	0.7310 (10)	0.4252 (8)	0.9513 (7)	0.0204 (14)	
N1	0.7737 (11)	0.4446 (7)	0.5575 (7)	0.0350 (15)	
H1	0.7413	0.5052	0.4768	0.042*	
C2	0.7787 (11)	0.3863 (7)	0.8109 (7)	0.0222 (14)	
C3	0.8671 (14)	0.2456 (9)	0.8087 (9)	0.0353 (18)	
H3	0.8970	0.1760	0.8954	0.042*	
C5	0.8633 (15)	0.3065 (10)	0.5567 (10)	0.044 (2)	
H5	0.8909	0.2816	0.4693	0.053*	
C4	0.9127 (16)	0.2052 (10)	0.6788 (10)	0.051 (3)	
H4	0.9756	0.1107	0.6775	0.061*	
O2	0.7138 (8)	0.3183 (5)	1.0690 (5)	0.0232 (10)	
O1W	0.9535 (9)	0.7979 (7)	0.7825 (6)	0.0398 (14)	
H1W	1.0327	0.7410	0.8417	0.060*	
H2W	1.0234	0.8615	0.7177	0.060*	
O2W	0.7275 (12)	0.5642 (8)	0.2640 (7)	0.0591 (19)	
H3W	0.6854	0.6442	0.2877	0.089*	
H4W	0.7313	0.5812	0.1725	0.089*	

### Atomic displacement parameters (Å^2^)

**Table:**

	*U*^11^	*U*^22^	*U*^33^	*U*^12^	*U*^13^	*U*^23^
Dy1	0.01446 (19)	0.01602 (18)	0.01286 (17)	−0.00079 (12)	−0.00214 (11)	−0.00175 (12)
O4	0.018 (3)	0.023 (2)	0.015 (2)	−0.007 (2)	−0.0030 (18)	0.0031 (18)
O1	0.029 (3)	0.020 (2)	0.017 (2)	−0.001 (2)	−0.0044 (19)	−0.0022 (19)
O7	0.046 (4)	0.029 (3)	0.027 (3)	0.009 (2)	−0.018 (2)	−0.011 (2)
C6	0.027 (4)	0.026 (4)	0.024 (4)	−0.006 (3)	0.000 (3)	−0.009 (3)
O3	0.038 (3)	0.025 (3)	0.019 (2)	0.003 (2)	−0.007 (2)	−0.008 (2)
C8	0.018 (4)	0.019 (3)	0.022 (3)	0.000 (3)	−0.002 (3)	−0.004 (3)
O6	0.035 (3)	0.023 (3)	0.026 (3)	0.008 (2)	−0.013 (2)	−0.009 (2)
C7	0.012 (4)	0.016 (3)	0.018 (3)	0.002 (3)	0.000 (2)	−0.004 (3)
O5	0.019 (3)	0.027 (3)	0.021 (2)	−0.006 (2)	−0.0065 (19)	0.005 (2)
C1	0.008 (3)	0.027 (4)	0.022 (3)	−0.006 (3)	−0.001 (2)	0.000 (3)
N1	0.041 (4)	0.037 (4)	0.028 (3)	−0.004 (3)	0.001 (3)	−0.015 (3)
C2	0.016 (4)	0.022 (3)	0.027 (4)	−0.003 (3)	0.001 (3)	−0.007 (3)
C3	0.036 (5)	0.029 (4)	0.038 (4)	−0.006 (4)	0.003 (4)	−0.009 (3)
C5	0.048 (6)	0.049 (5)	0.041 (5)	−0.006 (4)	0.006 (4)	−0.029 (4)
C4	0.063 (7)	0.034 (5)	0.053 (6)	−0.001 (5)	0.009 (5)	−0.021 (4)
O2	0.019 (3)	0.021 (2)	0.022 (2)	−0.001 (2)	−0.0043 (19)	0.0049 (19)
O1W	0.022 (3)	0.054 (4)	0.029 (3)	−0.010 (3)	−0.009 (2)	0.015 (3)
O2W	0.067 (5)	0.070 (5)	0.039 (4)	−0.019 (4)	−0.015 (3)	−0.004 (3)

### Geometric parameters (Å, °)

**Table:**

Dy1—O3	2.289 (5)	C7—O5	1.244 (8)
Dy1—O1W	2.352 (5)	C7—C7^ii^	1.549 (12)
Dy1—O2^i^	2.357 (5)	C1—O2	1.277 (8)
Dy1—O1	2.364 (4)	C1—C2	1.488 (9)
Dy1—O5^ii^	2.366 (4)	N1—C5	1.353 (11)
Dy1—O7^iii^	2.391 (5)	N1—H1	0.8600
Dy1—O6	2.402 (5)	C2—C3	1.377 (11)
Dy1—O4	2.415 (4)	C3—C4	1.398 (12)
O4—C7	1.247 (8)	C3—H3	0.9300
O1—C1	1.250 (8)	C5—C4	1.336 (13)
O7—C8	1.239 (8)	C5—H5	0.9300
C6—O3	1.275 (9)	C4—H4	0.9300
C6—N1	1.361 (9)	O1W—H1W	0.8500
C6—C2	1.424 (10)	O1W—H2W	0.8490
C8—O6	1.255 (8)	O2W—H3W	0.8534
C8—C8^iii^	1.547 (13)	O2W—H4W	0.8503
			
O3—Dy1—O1W	90.9 (2)	N1—C6—C2	115.8 (7)
O3—Dy1—O2^i^	90.55 (18)	C6—O3—Dy1	136.4 (4)
O1W—Dy1—O2^i^	148.35 (17)	O7—C8—O6	127.9 (6)
O3—Dy1—O1	73.20 (16)	O7—C8—C8^iii^	116.5 (7)
O1W—Dy1—O1	75.35 (18)	O6—C8—C8^iii^	115.6 (7)
O2^i^—Dy1—O1	74.84 (16)	C8—O6—Dy1	120.2 (4)
O3—Dy1—O5^ii^	81.96 (17)	O5—C7—O4	126.4 (6)
O1W—Dy1—O5^ii^	67.74 (17)	O5—C7—C7^ii^	116.8 (7)
O2^i^—Dy1—O5^ii^	143.60 (16)	O4—C7—C7^ii^	116.8 (7)
O1—Dy1—O5^ii^	134.77 (17)	C7—O5—Dy1^ii^	120.1 (4)
O3—Dy1—O7^iii^	148.30 (17)	O1—C1—O2	122.5 (6)
O1W—Dy1—O7^iii^	105.1 (2)	O1—C1—C2	120.4 (6)
O2^i^—Dy1—O7^iii^	89.70 (19)	O2—C1—C2	117.1 (6)
O1—Dy1—O7^iii^	136.90 (16)	C5—N1—C6	123.9 (7)
O5^ii^—Dy1—O7^iii^	79.21 (18)	C5—N1—H1	118.0
O3—Dy1—O6	144.68 (17)	C6—N1—H1	118.0
O1W—Dy1—O6	75.02 (19)	C3—C2—C6	119.6 (7)
O2^i^—Dy1—O6	85.84 (17)	C3—C2—C1	119.9 (7)
O1—Dy1—O6	71.95 (16)	C6—C2—C1	120.5 (6)
O5^ii^—Dy1—O6	119.90 (17)	C2—C3—C4	121.4 (8)
O7^iii^—Dy1—O6	66.91 (16)	C2—C3—H3	119.3
O3—Dy1—O4	77.17 (17)	C4—C3—H3	119.3
O1W—Dy1—O4	135.23 (16)	C4—C5—N1	121.4 (8)
O2^i^—Dy1—O4	75.68 (15)	C4—C5—H5	119.3
O1—Dy1—O4	137.50 (15)	N1—C5—H5	119.3
O5^ii^—Dy1—O4	67.92 (15)	C5—C4—C3	117.9 (8)
O7^iii^—Dy1—O4	72.20 (16)	C5—C4—H4	121.0
O6—Dy1—O4	134.95 (16)	C3—C4—H4	121.0
C7—O4—Dy1	118.2 (4)	C1—O2—Dy1^i^	128.1 (4)
C1—O1—Dy1	136.4 (4)	Dy1—O1W—H1W	125.7
C8—O7—Dy1^iii^	120.8 (4)	Dy1—O1W—H2W	124.6
O3—C6—N1	117.2 (6)	H1W—O1W—H2W	109.7
O3—C6—C2	127.0 (6)	H3W—O2W—H4W	109.6

Symmetry codes: (i) −*x*+1, −*y*+1, −*z*+2; (ii) −*x*+1, −*y*+2, −*z*+1; (iii) −*x*+1, −*y*+2, −*z*+2.

### Hydrogen-bond geometry (Å, °)

**Table:**

*D*—H···*A*	*D*—H	H···*A*	*D*···*A*	*D*—H···*A*
N1—H1···O2W	0.86	1.99	2.785 (9)	154
O1W—H1W···O2^iv^	0.85	1.94	2.732 (7)	155
O1W—H2W···O4^v^	0.85	2.07	2.751 (7)	137
O2W—H4W···O1^vi^	0.85	2.26	3.080 (8)	163
O2W—H4W···O6^vi^	0.85	2.36	2.878 (8)	120

Symmetry codes: (iv) −*x*+2, −*y*+1, −*z*+2; (v) *x*+1, *y*, *z*; (vi) *x*, *y*, *z*−1.
